# mRNA-1273 COVID-19 vaccine induces CD4+ T-cell responses among solid organ transplant recipients

**DOI:** 10.3389/fimmu.2025.1505871

**Published:** 2025-04-03

**Authors:** Bethany Girard, Amparo L. Figueroa, Stephen C. De Rosa, M. Juliana McElrath, Jamil R. Azzi, Dina Stolman, Uma Siangphoe, Elizabeth de Windt, Jacqueline M. Miller, Rituparna Das, Frances Priddy

**Affiliations:** ^1^ Moderna, Inc., Cambridge, MA, United States; ^2^ Division of Vaccine and Infectious Disease, Fred Hutchinson Cancer Center, Seattle, WA, United States; ^3^ Brigham and Women’s Hospital, Harvard Medical School, Boston, MA, United States

**Keywords:** cell-mediated immunity, COVID-19, mRNA-1273, solid-organ transplant recipients, vaccine

## Abstract

**Background:**

Cell-mediated immunity may provide durable protection against severe COVID-19, including among solid organ transplant recipients (SOTRs). This exploratory analysis in the open-label phase 3b trial evaluated cell-mediated immunity of mRNA-1273 in a subset of participants (59 kidney and 33 liver SOTRs; 12 immunocompetent participants).

**Methods:**

In Part A, SOTRs received three 100-µg doses of mRNA-1273; immunocompetent participants received two doses. In Part B, an additional 100-µg dose was offered ≥4 months after the primary series. SARS-CoV-2 spike (S) protein-specific T-cell responses were measured by intracellular cytokine staining and polyfunctionality analyses.

**Results:**

The primary series and additional dose of mRNA-1273 induced S protein-specific CD4^+^ T-cell responses exhibiting a Th-1-biased profile in both SOTRs and immunocompetent participants; however, response rates and magnitudes were lower among SOTRs. S protein-specific Th-2 CD4^+^ T-cell responses were below those observed for Th-1; CD8^+^ T-cell responses were not as robust among SOTRs compared with immunocompetent participants. Kidney SOTRs received multiple immunosuppressants and had lower cell-mediated immunity responses than liver SOTRs. Polyfunctional responses exhibited Th-1 cytokine signatures with ≤5 functional markers reported in SOTRs and immunocompetent participants.

**Conclusion:**

Overall, a three-dose mRNA-1273 primary series elicited Th-1-biased CD4^+^ T-cell responses among SOTRs that were improved with an additional dose.

**Clinical trial registration:**

https://beta.clinicaltrials.gov/study/NCT04860297?term=NCT04860297%20&rank=1, identifier NCT04860297.

## 1 Introduction

Solid organ transplant recipients (SOTRs) are at higher risk of severe COVID-19-related morbidity and mortality than immunocompetent individuals (1, 2). The use of post-transplant immunosuppressive therapies (ISTs) to prevent organ rejection and enhance graft function renders SOTRs highly susceptible to infection (3–5). The susceptibility of SOTRs to infection has been further highlighted during the COVID-19 pandemic, with increased rates of COVID-19-related outcomes that include severe disease, hospitalization, and death, compared with immunocompetent individuals (1, 2, 6–8). Vaccination against COVID-19 is an effective strategy to combat the risks associated with SARS-CoV-2 infections, including among SOTRs (9, 10). However, chronic immunosuppression in SOTRs may lead to suboptimal responses to vaccination, warranting the need for alternate vaccination strategies in this population (11–13).

COVID-19 vaccines mediate protection by eliciting binding and neutralizing antibody responses against the causative SARS-CoV-2 virus (14–16). In addition to humoral immune responses, COVID-19 vaccines also elicit cell-mediated immunity that can provide additional protection against severe COVID-19 caused by emergent SARS-CoV-2 variants of concern, which are less susceptible to neutralizing antibody-mediated immunity (17–20). In addition, more durable protection has been observed with cell-mediated immunity than with humoral immune responses, which tend to wane over time (16, 21, 22). Cell-mediated immune responses are indicative of robust immune memory in healthy adults with the magnitude of T-cell responses strongly correlating with a milder COVID-19 disease course (19). Post-transplant ISTs routinely used among SOTRs are known to reduce T-cell responses and may thereby compromise cell-mediated immune responses in this population (23, 24). Hence, vaccine strategies that induce humoral and cell-mediated immunity may be critical for effective protection against COVID-19 disease, especially among SOTRs.

The mRNA-1273 vaccine has been shown to elicit SARS-CoV-2 binding and neutralizing antibodies as well as inducing durable cell-mediated immune responses (25–27). Updated guidelines from the US Centers for Disease Control and Prevention recommend a three-dose primary series and additional dose(s) with a variant-containing mRNA vaccine for moderately and severely immunocompromised individuals, including SOTRs (28). In the phase 3b P304 trial (NCT04860297), the mRNA-1273 primary series (up to three 100-µg doses) was well tolerated and elicited humoral immune responses, which were enhanced following a 100-µg additional dose in SOTRs (29). To date, data on vaccine-induced cell-mediated immune responses among SOTRs are limited; however, a greater understanding is important for developing prevention strategies that induce T-cell responses against COVID-19 and can provide durable protection against SARS-CoV-2 infection. Here, we present cell-mediated immunity data in a subset of kidney and liver SOTRs who received a three-dose mRNA-1273 primary series and immunocompetent participants who received a two-dose mRNA-1273 primary series (Part A) and a single 100-µg additional dose (Part B) in the P304 trial.

## 2 Materials and methods

### 2.1 Study design and participants

The open-label, phase 3b study (NCT04860297) enrolled kidney and liver SOTRs as well as immunocompetent individuals at 16 sites in the United States and was conducted in two parts. In Part A, kidney and liver SOTRs received a three-dose mRNA-1273 primary vaccination series; immunocompetent individuals received a two-dose primary series. In Part B, SOTRs and immunocompetent participants rolled over from Part A, along with additional kidney and liver SOTRs who had completed the primary series outside of the study, received an additional dose of mRNA-1273. The cell-mediated immunity analysis was conducted in a subset of SOTRs and immunocompetent participants who were baseline SARS-CoV-2 seronegative and had sufficient cell numbers for analysis. Data were collected between April 16, 2021, and September 1, 2022 (data cutoff).

Eligible participants for Part A were aged ≥18 years, had undergone kidney or liver transplantation ≥90 days prior to enrollment, and had received chronic IST for the prevention of allograft rejection for ≥90 days. Immunocompetent participants who were in good health, aged ≥18 years, and had not received any COVID-19 vaccine were eligible. In Part B, eligible participants included those who participated in Part A and were at least ≥4 months from the last dose. Eligible participants were aged ≥18 years, had received a kidney or liver transplant ≥90 days prior to enrollment, and had completed a primary COVID-19 vaccination series (three doses of an mRNA vaccine, two doses of a non-mRNA vaccine, or more than one dose of non-mRNA vaccine combined with one dose of mRNA vaccine) after receipt of transplant. SOTRs who had completed a primary series outside of the study after transplant were also included in Part B.

### 2.2 Ethics statement

Written informed consent was obtained from all participants prior to the initiation of the study. This study was conducted in accordance with the International Council for Harmonisation of Technical Requirements for Pharmaceuticals for Human Use, Good Clinical Practice guidelines, and all applicable regulatory requirements. Study materials including the protocol, amendments (protocol number: mRNA-1273-P304), and the informed consent forms were approved by an institutional review board (IRB; Advarra IRB identification number: Pro00051035).

### 2.3 Trial vaccine

**Figure 1:**
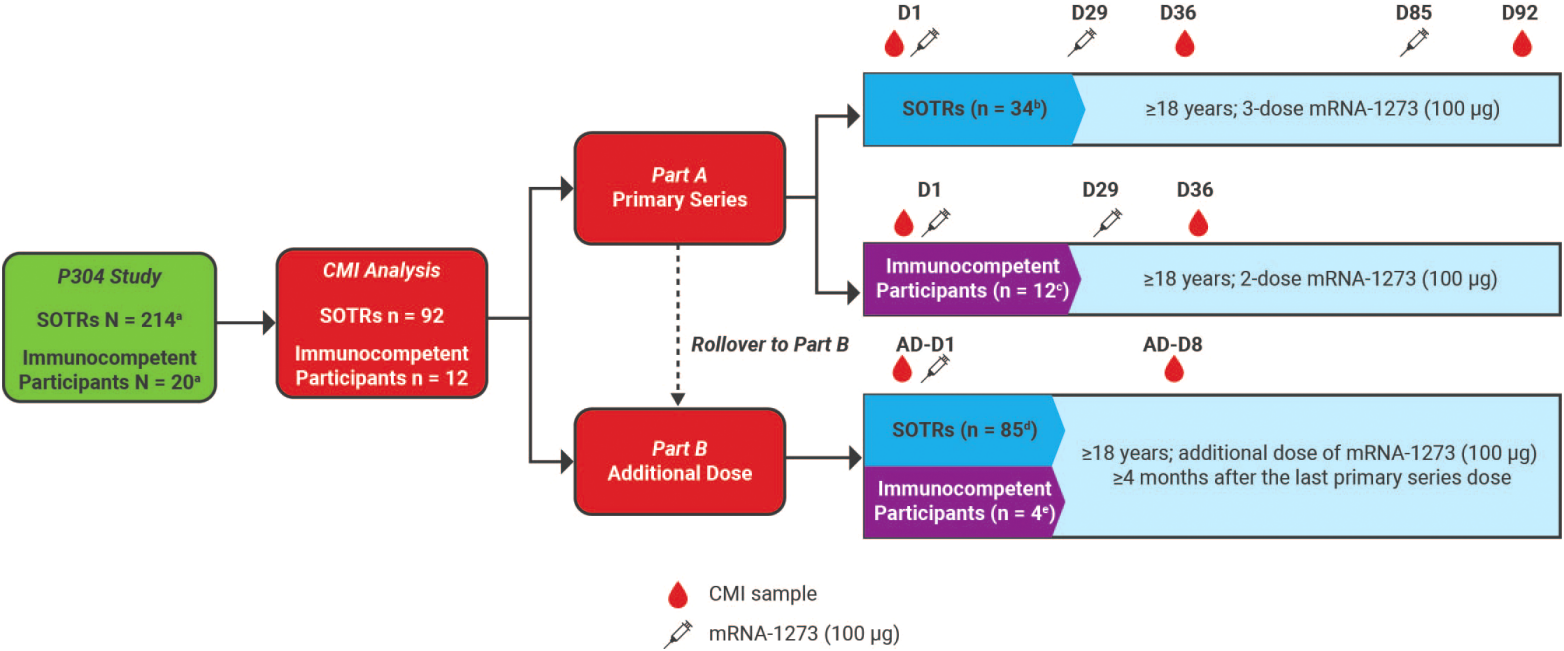
CMI analysis overview. In Part A, immunocompetent participants received two doses of mRNA-1273 on Days 1 and 29; unvaccinated SOTRs received dose 3 on Day 85 (56 days post dose 2). Additionally, SOTRs in Part A vaccinated with two doses of mRNA-1273 prior to the study received dose 3 on Day 1. In Part B, immunocompetent participants vaccinated with two doses of mRNA-1273 and SOTRs who completed primary mRNA or non-mRNA vaccination received an additional dose on Day 1. Peripheral blood samples were collected pre-dose (Day 1), post dose 2 (Day 36), post dose 3 (Day 92), and post additional dose (Day 8 post additional dose). Samples were collected on Day 8 post dose 3 from a subset of SOTRs who received two doses of mRNA-1273 outside of the study; these samples were combined with post dose 3 samples for analysis. At each time point, only samples with sufficient cell viability and cell count were included for CMI analysis. ^a^Number of participants enrolled in the P304 study. ^b^Part A SOTRs: participants with available samples included in the analysis post dose 3 (n = 34). ^c^Part A immunocompetent: participants with evaluable samples for analysis post dose 2 (n = 12). ^d^Part B SOTRs: participants who received an additional dose of mRNA-1273, including participants who rolled over from Part A (n = 27) and participants who only received an additional dose in the study (n = 58). ^e^Part B immunocompetent: participants rolled over from Part A with evaluable samples for analysis post additional dose of mRNA-1273 (n = 4). AD, additional dose; CMI, cell-mediated immunity; D, day; SOTR, solid organ transplant recipient.

mRNA-1273 is an mRNA-lipid nanoparticle vaccine encoding the stabilized prefusion S protein of SARS-CoV-2. mRNA-1273 was provided as a 0.2 mg/mL suspension and was administered intramuscularly into the deltoid muscle as a 0.5-mL dose containing 100 µg of mRNA. In Part A, unvaccinated SOTRs and immunocompetent participants who consented to two doses received mRNA-1273 on Days 1 and 29; unvaccinated SOTRs who consented to three doses received the third dose on Day 85 (56 days post dose 2). SOTRs in Part A who were previously vaccinated with two doses of mRNA-1273 prior to the study received a third dose on Day 1 (≥1 month from the last dose) (**Figure 1**). In Part B, immunocompetent participants vaccinated with two doses of mRNA-1273 and SOTRs who completed the primary vaccination series with mRNA or non-mRNA COVID-19 vaccines (≥4 months from the last dose) received an additional dose on Day 1.

### 2.4 Study objectives and endpoints

The objective of this exploratory analysis was to assess the SARS-CoV-2 S protein-specific T-cell immune responses in a subset of SOTRs and immunocompetent participants. The exploratory endpoints were phenotype and percentage of cytokine-expressing S protein-specific T cells (response magnitude) as well as the proportion of participants with responses determined to be positive (response rate), as measured by flow cytometry at different time points after vaccination in SOTRs and immunocompetent participants.

### 2.5 Peripheral blood mononuclear cell sample processing

In Part A, peripheral blood mononuclear cells (PBMCs) from SOTRs were collected pre-dose (Day 1), 7 days post dose 2 (Day 36), and 7 days post dose 3 (Day 92). Samples were also collected at dose 3 (Day 1) and post dose 3 (Day 8) from a subset of SOTRs who received two doses of mRNA-1273 outside of the study; these samples were combined with post dose 3 samples for analysis. For immunocompetent participants, samples were collected at pre-dose (Day 1) and 7 days post dose 2 (Day 36). In Part B, blood samples were collected at pre additional dose (AD-D1) and post additional dose [Day 8 post additional dose (AD-D8)]. Blood samples were collected from a subset of participants in ethylenediaminetetraacetic acid tubes and shipped to a central laboratory for processing. Blood samples were transferred into SepMate tubes and centrifuged at 1,200 ×*g* for 20 minutes at room temperature. Thereafter, the mononuclear cell layer was harvested, transferred to a 50-mL conical tube, and washed twice with Ca^+2^, Mg^+2^-free phosphate-buffered solution. Live cells were counted using an automated cell counter and cryopreserved at 10 million cells/mL per tube in a CoolCell or Mr. Frosty. Cells were stored in liquid nitrogen until shipped for analysis.

### 2.6 Intracellular cytokine staining assay

The phenotype and percentage of SARS-CoV-2 S protein-specific CD4^+^ and CD8^+^ T-cell responses were measured using a validated intracellular cytokine staining (ICS) assay, as described previously (30, 31). The details of the staining panel used in the ICS assay are included in **Supplementary Table 1**. Peptide pools covering the spike protein of SARS-CoV-2 (316 peptides with four peptides covering the D614G variant) were synthesized as 15 amino acids overlapping with 11 amino acids (Bio-Synthesis, Lewisville, TX, USA). The peptides were pooled into two pools (S1, 173 peptides; and S2, 147 peptides) and were used for the 6-hour stimulation. Both peptide pools were used at a final concentration of 1 µg/mL for each peptide. Dimethyl sulfoxide diluent was included as a negative control. Cells stimulated with a polyclonal stimulant, staphylococcal enterotoxin B, were included as a positive control. Samples were analyzed using a BD FACSymphony A5 flow cytometer (BD Biosciences, San Jose, CA, USA) with a five-laser configuration. Data were analyzed in a blinded fashion using standardized templates in FlowJo v.9.9.4 (BD Biosciences). Gating details are provided in the **Supplementary Material** (**Supplementary Table 1**, **Supplementary Figure 1**).

### 2.7 Polyfunctional analysis

The Combinatorial Polyfunctionality Analysis of Antigen-Specific T-Cell Subsets (COMPASS) utilizes a Bayesian hierarchical mixture model to identify antigen-specific changes in all T-cell subsets simultaneously (32). This model was used to analyze CD4^+^ and CD8^+^ T-cell responses to S1 and S2 peptide pools, with the magnitude of antigen-specific responses. Bar graphs and heatmaps were developed to show different cell subsets expressing one or more cytokines/functional markers.

### 2.8 Statistical analysis

The positivity for a peptide pool within a T-cell subset was assessed by constructing a two-by-two contingency table to compare the peptide-stimulated and negative control data for each cytokine subset. The four entries in each table represented the numbers of cells that were positive and negative for the cytokine(s) for both the peptide-stimulated and negative control data. If both negative control replicates were included, the average for the total number of cells and the average for the number of positive cells were used. A one-sided Fisher’s exact test was applied to determine whether the number of cytokine-producing cells for the peptide-stimulated data was equal to that for the negative control. Multiple individual tests were conducted simultaneously for each peptide pool; therefore, a multiplicity adjustment was made to the p-values of individual peptide pools using the Bonferroni–Holm adjustment method. If the adjusted p-value for a peptide pool was ≤0.00001, the response to the peptide pool for the T-cell subset was considered positive. Due to the large total cell counts for the T-cell subsets (e.g., <100,000 cells), Fisher’s exact test had high power to reject the null hypothesis for very small differences. Therefore, the adjusted p-value significance threshold was chosen stringently (≤0.00001) (31). The overall response to spike protein was considered positive if ≥1 spike peptide pool (S1 or S2) was positive for the T-cell subset.

## 3 Results

### 3.1 Participants

Overall, a total of 214 SOTR and 20 immunocompetent participants were enrolled for the P304 study and received mRNA-1273 vaccine doses (100 μg). The exploratory cell-mediated immunity analysis was conducted on PBMCs collected from a subset of 104 participants who were baseline SARS-CoV-2 seronegative and had sufficient cell numbers for analysis (kidney SOTRs, n = 59; liver SOTRs, n = 33; immunocompetent participants, n = 12; **Table 1**). In Part A, 34 SOTRs received three doses of mRNA-1273, and 12 immunocompetent participants received two doses (**Figure 1**). In Part B, 85 previously vaccinated SOTRs and four immunocompetent participants from Part A received an additional dose (**Figure 1**). Four SOTRs who were included in the additional dose analysis were noted to have tested positive via reverse transcriptase–polymerase chain reaction; however, these participants had no antibody conversion (early infection) before the additional dose, so they were considered as seronegative and were included in the analysis.

**Table 1:** Summary of baseline characteristics and demographics of study participants included in the exploratory analysis.

Characteristic	SOTRs			Immunocompetent participants (N = 12)
	Total (N = 92)	Kidney (n = 59)	Liver (n = 33)	
Mean age at screening, years (SD)	54.0 (13.8)	55.2 (14.0)	51.8 (13.5)	47.9 (15.3)
Female, n (%)	44 (47.8)	27 (45.8)	17 (51.5)	9 (75.0)
Race, n (%)				
White	68 (73.9)	44 (74.6)	24 (72.7)	7 (58.3)
Black/African American	16 (17.4)	10 (17.0)	6 (18.2)	0
Asian	3 (3.3)	3 (5.1)	0	2 (16.7)
American Indian or Alaska Native	0	0	0	1 (8.3)
Multiple	2 (2.2)	1 (1.7)	1 (3.0)	0
Other	1 (1.1)	0	1 (3.0)	1 (8.3)
Not reported	1 (1.1)	1 (1.7)	0	0
Unknown	1 (1.1)	0	1 (3.0)	1 (8.3)
Ethnicity, n (%)				
Hispanic or Latino	8 (8.7)	5 (8.5)	3 (9.1)	1 (8.3)
Not Hispanic or Latino	84 (91.3)	54 (91.5)	30 (90.9)	11 (91.2)
Previous induction therapy, n (%)				
Basiliximab	23 (25.0)	21 (35.6)	2 (6.1)	–
Thymoglobulin	18 (19.6)	16 (27.1)	2 (6.1)	–
Other^a^	41 (44.6)	20 (33.9)	21 (63.6)	–
Concomitant IST, n (%)^b^				
Antiproliferatives	73 (70.2)	54 (91.5)	19 (57.6)	–
Calcineurin inhibitors	86 (82.7)	53 (89.8)	32 (97.0)	–
mTOR inhibitors	4 (3.9)	2 (3.4)	2 (6.1)	–
Steroids	56 (53.9)	44 (74.6)	12 (36.4)	–
Other	8 (7.7)	5 (8.5)	3 (9.0)	–
Concomitant combination IST, n (%)^c^				
Mycophenolate, tacrolimus	21 (22.8)	10 (17.0)	11 (33.3)	–
Mycophenolate, tacrolimus, prednisone	29 (31.5)	28 (47.5)	1 (3.0)	–
Tacrolimus	10 (10.9)	0	10 (30.3)	–
Other	32 (34.8)	21 (35.6)	11 (33.3)	–
Time since transplantation				
Mean, years	6.9			
Median, years	4.4			
<2 years, n (%)	20 (21.7)			
≥2 years, n (%)	72 (78.3)			

Vaccination status of individuals included in the CMI analysis, n				
	SOTRs			Immunocompetent participants
	Total	Kidney	Liver	
Part A (primary series)^d,e^				
N	34	20	14	12
Immunocompetent participants who received a total of 2 doses in the study	–	–	–	12
SOTRs who received a total of 3 doses in study (previously unvaccinated)	19	6	13	–
SOTRs who received dose 3 only in study (previously vaccinated)	15	14	1	–
Part B (additional dose)^e^				
N	92	59	33	8
Immunocompetent participants who received a 2-dose primary series in Part A and received a third additional dose in Part B				8
SOTRs who received or completed primary series in study (Part A rollover)	34	20	14	–
SOTRs who received primary series outside of study	58	39	19	–

CMI, cell-mediated immunity; IST, immunosuppressive therapy; mTOR, mechanistic target of rapamycin; PBMCs, peripheral blood mononuclear cells; SD, standard deviation; SOTRs, solid organ transplant recipients.

a Previous induction therapy, other: alemtuzumab, daclizumab, methylprednisolone, prednisone, a combination with basiliximab or thymoglobulin.

b IST types included antiproliferative (mycophenolate mofetil, mycophenolate sodium, mycophenolic acid, or azathioprine), calcineurin inhibitors (tacrolimus or cyclosporine), mTOR inhibitors (sirolimus or everolimus), steroids (budesonide, methylprednisolone, and prednisone), and others (adalimumab, belatacept, hydroxychloroquine, infliximab, and capecitabine).

c Concomitant combination IST, other: combinations of tacrolimus, azathioprine, and prednisone; mycophenolate and prednisone; tacrolimus and azathioprine; and other rare combinations.

d Three-dose primary series for SOTRs; 2-dose primary series for immunocompetent participants.

e PBMCs were collected from a subset of participants for inclusion in the exploratory analysis; samples with sufficient cell numbers were included in the CMI analysis.

Baseline characteristics and demographics of study participants in this exploratory analysis are shown in **Table 1**. Among SOTRs, 73.9% of participants were White, 52.2% were male, and 64.1% were kidney transplant recipients. The median time post-transplantation was 4.4 years. Concomitant combination IST with mycophenolate, tacrolimus, and prednisone was more commonly received among kidney (47.5%) than liver (3.0%) SOTRs, whereas tacrolimus monotherapy was more common among liver (30.3%) SOTRs than kidney SOTRs (0%). Previous induction therapy with basiliximab and thymoglobulin was more commonly received among kidney SOTRs (35.6% and 27.1%, respectively) than liver SOTRs (6.1% for both induction therapies). Most immunocompetent participants were female (75.0%) and White (58.3%).

### 3.2 SARS-CoV-2 S-specific T-cell responses to mRNA-1273 vaccination

**Figure 2:**
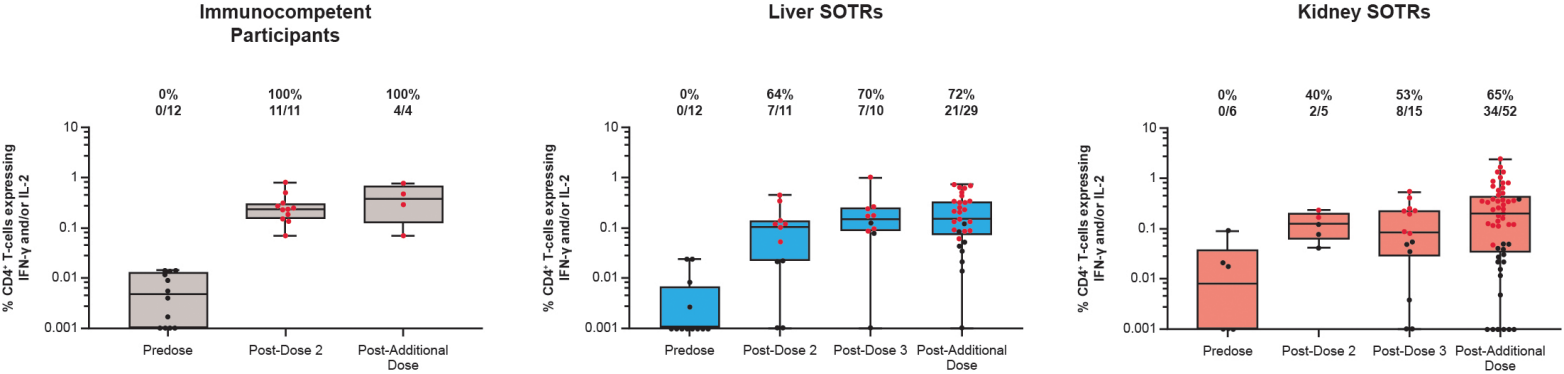
Th-1-biased CD4^+^ T-cell responses to SARS-CoV-2 S protein among SARS-CoV-2-seronegative participants who received up to four doses of mRNA-1273 in the P304 trial. CD4^+^ T-cell responses to SARS-CoV-2 S protein exhibiting Th-1 profile (IFN-γ and/or IL-2) among SARS-CoV-2-seronegative participants who received up to four doses (100 μg) of mRNA-1273 in the P304 trial as measured by flow cytometry. Response rates based on the number and percentage of participants with responses to S1 and S2 peptide pools are shown above each bar on the graph. Positive responses (denoted in red) were determined using Fisher’s exact test comparing responses in the peptide pool stimulation versus the negative control (dimethyl sulfoxide, the peptide diluent). Horizontal lines within boxes indicate median values; vertical bars span the lower quartile (the minimum) and the upper quartile (the maximum). IFN, interferon; IL, interleukin; SOTR, solid organ transplant recipient.

S-specific CD4^+^ and CD8^+^ T-cell responses to the mRNA-1273 primary vaccination and an additional dose in SOTRs and immunocompetent individuals were assessed using an ICS assay following 6-hour stimulation with S1 and S2 peptide pools. Results are shown as the percentage of the CD4^+^ or CD8^+^ T cells expressing the cytokine(s) of interest (response magnitude) as well as the response rate determined by the percentage of participants with positive responses (based on statistical comparison to the negative control stimulation condition). In Part A, T helper type 1 (Th-1)-biased CD4^+^ responses [expressing interferon (IFN)-γ and/or interleukin (IL)-2] were not present among SOTRs and immunocompetent individuals at pre-vaccination (Day 1; **Figure 2**, **Supplementary Table 2**). All immunocompetent participants exhibited Th-1 CD4^+^ T-cell responses at Day 36 (7 days post dose 2); vaccinated SOTR participants had increased magnitudes of these responses, although, based on our positivity definition, not all were deemed positive (64% and 40% response rates for liver SOTR and kidney SOTRs, respectively). These responses were maintained among SOTRs to Day 92 (7 days post dose 3) with an increase in response rates to 70% for liver SOTR and 53% for kidney SOTR.

In Part B, an additional dose of mRNA-1273 further increased S protein-specific CD4^+^ T-cell responses that exhibited a Th-1-biased profile in both SOTRs and immunocompetent participants (**Figure 2**, **Supplementary Table 2**). All immunocompetent participants (100%) exhibited Th-1 CD4^+^ T-cell response rates following an additional dose of mRNA-1273. The proportion of SOTRs with positive Th-1 CD4^+^ T-cell response rates increased after the additional dose of mRNA-1273 (72% and 65% for liver and kidney SOTRs, respectively). For both liver and kidney SOTRs, similar magnitudes of response were maintained post dose 3 and post additional dose.

**Figure 3:**
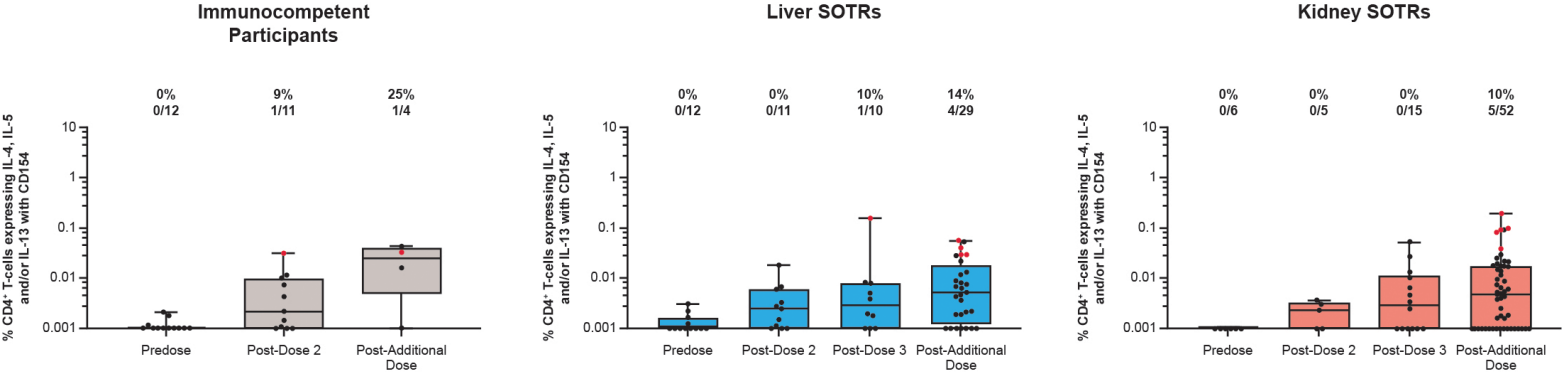
Th-2 CD4^+^ T-cell responses to SARS-CoV-2 S protein among SARS-CoV-2-seronegative participants who received up to four doses of mRNA-1273 in the P304 trial. CD4^+^ T-cell responses to SARS-CoV-2 S protein expressing IL-4 and/or IL-5 and/or IL-13 and CD154 among SARS-CoV-2-seronegative participants who received up to four doses (100 μg) of mRNA-1273 in the P304 trial as measured by flow cytometry. Response rates based on the number and percentage of participants with responses to S1 and S2 peptide pools are shown above each bar on the graph. Positive responses (denoted in red) were determined using Fisher’s exact test comparing responses in the peptide pool stimulation versus the negative control (dimethyl sulfoxide, the peptide diluent). Horizontal lines within boxes indicate median values; vertical bars span the lower quartile (the minimum) and the upper quartile (the maximum). IFN, interferon; IL, interleukin; SOTR, solid organ transplant recipients.

T-helper type 2 (Th-2) CD4^+^ T-cell responses (expressing IL-4, IL-5, and/or IL-13 with CD154 co-expression) are represented in **Figure 3**. Prior to vaccination, CD4^+^ Th-2 response rates were not detected in SOTRs and immunocompetent participants (**Figure 3**, **Supplementary Table 2**). The percentage of S protein-specific CD4^+^ T cells expressing Th-2 cytokines as well as the response rate for these responses were lower than Th-1-biased responses after mRNA-1273 vaccination in both SOTRs and immunocompetent participants (**Figure 3**). Vaccination of SOTRs and immunocompetent individuals with a primary series and additional dose did not substantially induce CD4^+^ Th-2 responses throughout the study.

**Figure 4:**
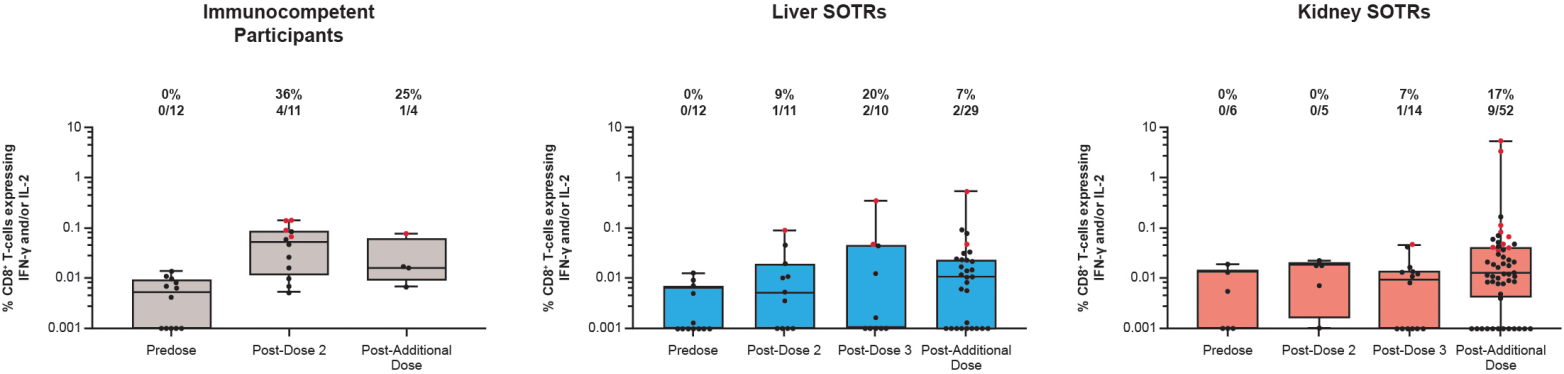
CD8^+^ T-cell responses to SARS-CoV-2 S protein among SARS-CoV-2-seronegative participants who received up to four doses of mRNA-1273 in the P304 trial. CD8^+^ T-cell responses to SARS-CoV-2 S protein expressing IFN-γ and/or interleukin-2 among SARS-CoV-2-seronegative participants who received up to four doses (100 μg) of mRNA-1273 in the P304 trial as measured by flow cytometry. Response rates based on the number and percentage of participants with responses to S1 and S2 peptide pools are shown above each bar on the graph. Positive responses (denoted in red) were determined using Fisher’s exact test comparing responses in the peptide pool stimulation versus the negative control (dimethyl sulfoxide, the peptide diluent). Horizontal lines within boxes indicate median values; vertical bars span the lower quartile (the minimum) and the upper quartile (the maximum). IFN-γ, interferon gamma; IL, interleukin; SOTRs, solid organ transplant recipients.

The cellular immune response among SOTRs and immunocompetent individuals was further evaluated by measuring the S protein CD8^+^ T-cell responses expressing IFN-γ or IL-2. At pre-vaccination, CD8^+^ T-cell responses were not detected among SOTRs and immunocompetent individuals (**Figure 4**). Similar to CD4^+^ T-cell responses, there was an increase in the magnitude of CD8^+^ IFN-γ^+^ and/or IL-2^+^ responses after vaccination in SOTRs; however, these positive responses were not as robust when compared with those in immunocompetent individuals (**Figures 3**, **4**). Following dose 2, 36% of immunocompetent individuals exhibited positive response rates, while only 9% of liver SOTRs and 0% of kidney SOTRs had detectable response rates. Following the additional dose of mRNA-1273, CD8^+^ T-cell responses expressing IFN-γ and/or IL-2 were reduced but remained detectable among liver SOTRs and increased for kidney SOTRs with a response rate of 17% and magnitudes similar to those seen in immunocompetent participants (**Figure 4**, **Supplementary Table 2**).

### 3.3 Polyfunctional T-cell responses to mRNA-1273 vaccination

Polyfunctionality refers to the ability of T cells to perform a diversity of functions, such as the concomitant expression of an array of cytokines, chemokines, or cytotoxic granules at the cellular level (33). Polyfunctionality of CD4^+^ T-cell responses to S1 and S2 peptide pools was evaluated in SOTRs and immunocompetent individuals using COMPASS (32).

**Figure 5:**
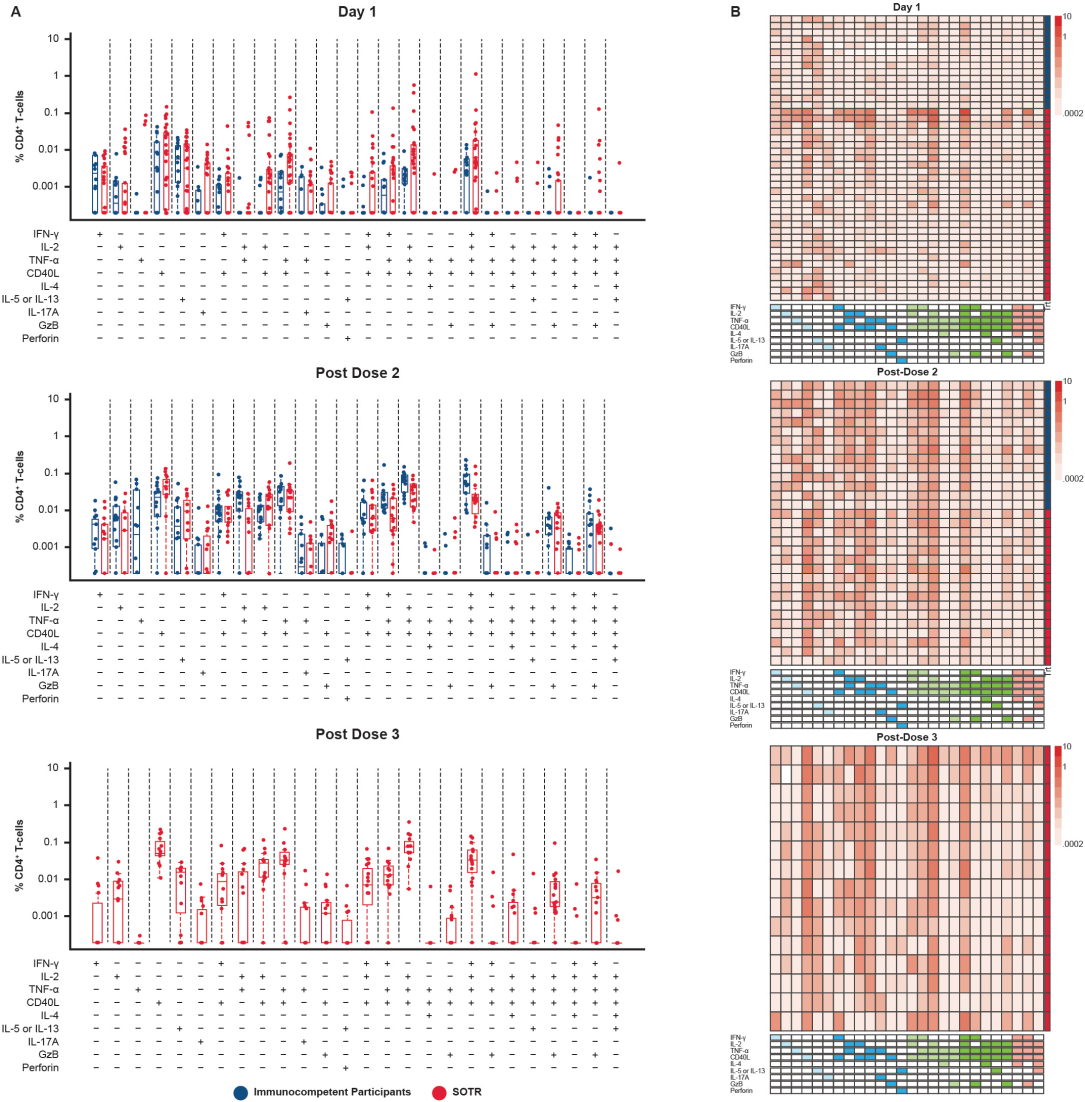
CD4^+^ T-cell polyfunctionality among SARS-CoV-2-seronegative participants using S1+S2 peptide pool in SOTRs after two and three doses of mRNA-1273 in the P304 trial. Polyfunctional CD4^+^ T-cell responses to S1 and S2 peptide pools were assessed using COMPASS (32). **(A)** Bar graphs represent different cell subsets expressing one or more cytokines/functional markers. Each bar shows the magnitude of responses of the corresponding antigen-specific subset being expressed. SOTRs (n = 34, red dots) received a three-dose primary series and additional dose of mRNA-1273 (100 μg); immunocompetent participants (n = 12; blue dots) received a two-dose primary series and an additional dose of mRNA-1273 (100-µg). **(B)** Heatmaps are shown next to each bar plot. Columns correspond to different cell subsets expressing one or more cytokines/functional markers, color-coded and ordered by degree of functionality from one function on the left (light blue boxes) to five functions on the right (pink boxes) in the key below the heatmap; a filled box indicates that cells in that column are expressing that function, with different colors indicating cells with one, two, three, four, or five functions. Rows correspond to the individual SOTR (n = 34; red boxes) and immunocompetent (n = 12; blue boxes) participants in each treatment group at each time point. Each cell shows the magnitude of responses of the corresponding antigen-specific subset (column) being expressed (row) and is color-coded ranging from white to dark red. The column on the far right of each heatmap indicates the group that the participant in each row belongs to (blue, immunocompetent; red, SOTR). COMPASS, Combinatorial Polyfunctionality Analysis of Antigen-Specific T-cell Subsets; GzB, Granzyme B; IFN-γ, interferon gamma; IL, interleukin; SOTRs, solid organ transplant recipients; TNF-α, tumor necrosis factor alpha.

**Figure 6:**
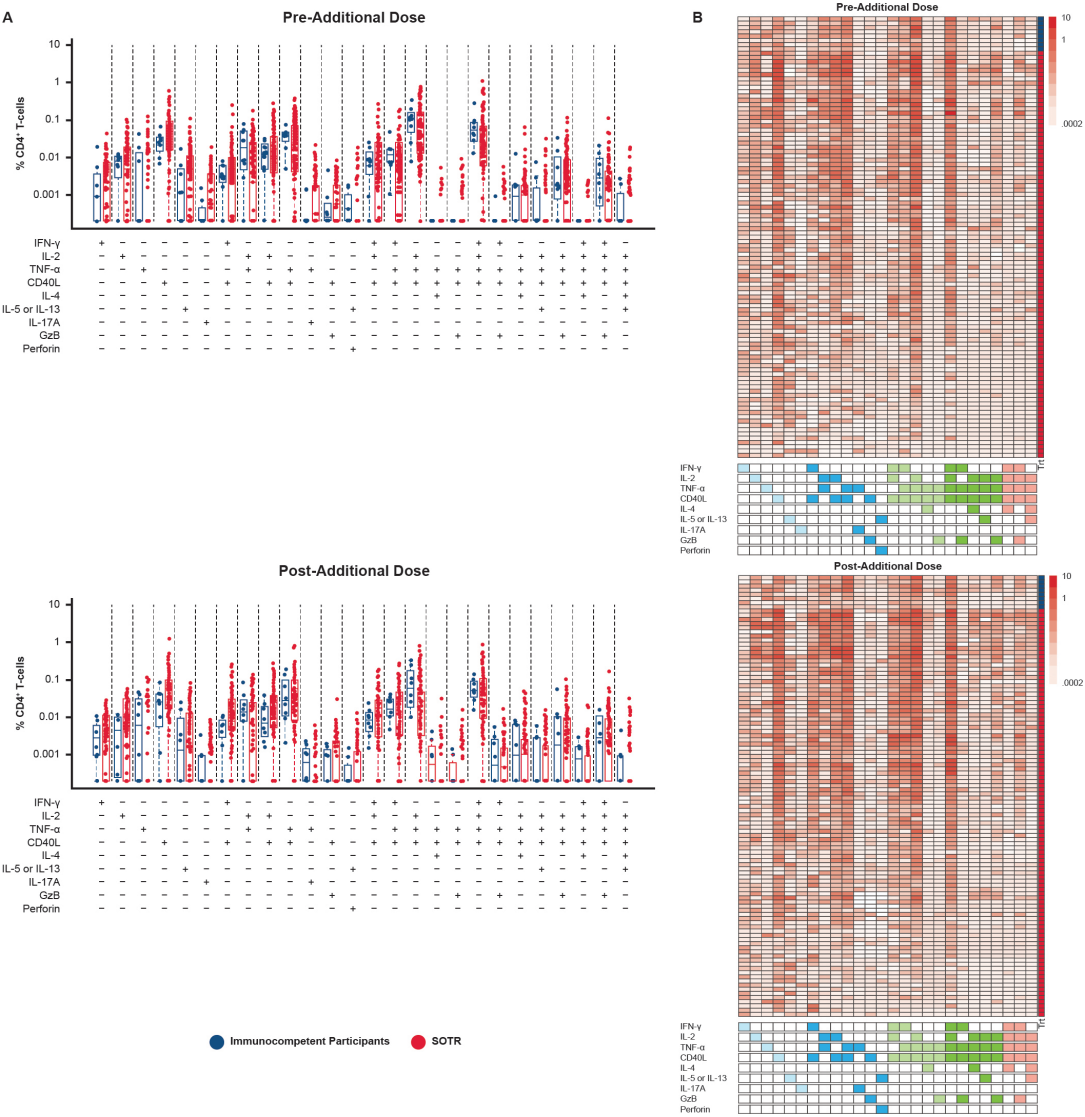
CD4^+^ T-cell polyfunctionality among SARS-CoV-2-seronegative participants using S1+S2 peptide pool in SOTR pre and post additional dose of mRNA-1273 in the P304 trial. Polyfunctionality analysis was conducted using COMPASS (32). **(A)** Bar graphs represent different cell subsets expressing one or more cytokines/functional markers. Each bar shows the magnitude of responses of the corresponding antigen-specific subset being expressed. SOTRs (n = 85; red dots) received a three-dose primary series and additional dose of mRNA-1273 (100 µg); immunocompetent participants (n = 4; blue dots) received a two-dose primary series and an additional dose of mRNA-1273 (100 µg). **(B)** Heatmaps are shown next to each bar plot. Columns correspond to different cell subsets expressing one or more cytokines/functional markers, color-coded and ordered by degree of functionality from one function on the left (light blue boxes) to five functions on the right (pink boxes) in the key below the heatmap; a filled box indicates that cells in that column are expressing that function, with different colors indicating cells with one, two, three, four, or five functions. Rows correspond to the individual SOTR (n = 85; red boxes) and immunocompetent (n = 4; blue boxes) participants in each treatment group at each time point. Each cell shows the magnitude of responses of the corresponding antigen-specific subset (column) being expressed (row) and is color-coded ranging from white to dark red. The column on the far right of each heatmap indicates the group that the participant in each row belongs to (blue, immunocompetent; red, SOTR). COMPASS, Combinatorial Polyfunctionality Analysis of Antigen-Specific T-cell Subsets; GzB, Granzyme B; IFN-γ, interferon gamma; IL, interleukin; SOTRs, solid organ transplant recipients; TNF-α, tumor necrosis factor alpha.

Expression of a combination of the nine functional markers examined was observed in many CD4^+^ T cells, including co-expression of up to five of these markers. IFN-γ, IL-2, tumor necrosis factor (TNF)-α, and CD40L were among the functional markers most commonly detected in both SOTRs and immunocompetent participants after two doses of mRNA-1273, demonstrating a Th-1-skewed response to spike protein antigens after vaccination in both populations (**Figures 5**, **6**). Polyfunctionality analysis demonstrated a highly diverse functional profile among CD4^+^ T-cell responses to S1 and S2 peptide pools among SOTRs and immunocompetent participants at pre-dose and 1 month post additional dose of mRNA-1273 (**Figure 6**). Similar Th-1 cytokine signatures were observed regardless of SOTR versus immunocompetent status, with polyfunctional responses consisting of ≤5 functional markers expressed in both groups. Polyfunctional expression of the nine functional markers in CD8^+^ T cells was lower in magnitude compared with that in CD4^+^ T cells; however, this expression was comparable between immunocompetent individuals and SOTRs (**Supplementary Figure 2**).

## 4 Discussion

This article presents exploratory findings on cell-mediated immunity elicited by the SARS-CoV-2 mRNA-1273 primary series and additional dose from an open-label phase 3b trial in SOTRs and immunocompetent participants. Previous results from Parts A and B of this trial demonstrated that a three-dose primary series and additional dose had an acceptable safety profile and boosted humoral immune responses, particularly among liver SOTRs (29). To expand on these results, the current analysis employed ICS and T-cell polyfunctionality analyses to assess SARS-CoV-2 S-specific immune responses in SOTRs and immunocompetent individuals.

Overall, the three-dose mRNA-1273 (100-μg) primary series elicited Th-1-biased CD4^+^ T-cell responses among liver and kidney SOTRs that were enhanced by an additional dose (100 μg), suggesting that cell-mediated immunity is improved by additional vaccine doses in SOTRs and supports an enhanced SARS-CoV-2-specific immune response in immunocompromised groups. These responses, although lower in magnitude, were consistent with the CD4^+^ T-cell response rates observed among immunocompetent individuals. Polyfunctional CD4^+^ T-cell responses to mRNA-1273 were also observed in SOTRs, with similar Th-1 cytokine signatures observed among SOTRs and immunocompetent individuals; polyfunctional responses consisting of up to five functional markers were reported for both groups. CD8^+^ T-cell response rates, following the primary series and additional dose, were generally lower in SOTRs than in immunocompetent individuals. While a similar pattern was observed among healthy adults in previous studies in which robust CD4^+^ Th-1-biased responses were evident, albeit with a less robust CD8^+^ response (26, 34), the data presented here imply that SOTRs are potentially even less capable of mounting a CD8^+^ T-cell response following vaccination compared with immunocompetent individuals.

The lower cell-mediated immune responses observed among SOTRs compared with immunocompetent individuals may be attributed to the use of ISTs. A major barrier to successful organ transplantation is the recognition of non-self major histocompatibility complex on transplant organs, which activates T cells and, therefore, requires ISTs to prevent graft rejection (5, 35, 36). ISTs target T-cell responses to circumvent allograft rejection (36, 37). Consequently, routine ISTs among SOTRs present an additional hurdle for cell-mediated immunity for this population (36, 37). The majority of SOTRs (89.9% kidney and 97.0% liver) in the current analysis were in receipt of calcineurin inhibitors, namely, tacrolimus or cyclosporine, which are known to reduce cytokine production and decrease T-cell proliferation (36, 38, 39), thereby reducing cell-mediated immunity among this population. Notably, while CD4^+^ T-cell responses were lower in magnitude in SOTRs compared with immunocompetent individuals following two doses of mRNA-1273, response rates increased among SOTRs following a third dose and additional dose.

COVID-19 vaccines, including mRNA-1273, have been updated to target emergent variants to maintain protection against SARS-CoV-2 infection and are authorized for use among SOTRs. While humoral responses are major drivers of vaccine-mediated immunity against COVID-19, antibody levels wane over time (16, 22). The emergence of SARS-CoV-2 variants that can evade antibodies elicited through prior infection or vaccination has spurred great interest in exploring cell-mediated immunity as a means to further enhance immunity (18, 20). Vaccination against COVID-19 can activate SARS-CoV-2-specific T-cell responses that can be maintained for a long period of time, thereby providing more resistance to emerging variants than with humoral immune responses alone (18, 20).

Based on the findings of this analysis, mRNA-1273 demonstrates the potential to enhance CD4^+^ cell-mediated immune responses among SOTRs. A three-dose primary series and additional dose (100 μg) elicited a Th-1-biased CD4^+^ T-cell immune response post dose 3 with further increases after the additional dose in SOTRs. CD4^+^ T cells are involved in inducing immune responses and are a critical component of immune protection; memory T cells play a vital role in viral clearance and prevent or limit re-infection by producing high-affinity antibody responses while modulating CD8^+^ T-cell responses (40). Notably, CD4^+^ T-cell responses among liver transplant recipients were higher than those observed among kidney organ transplant recipients. The majority of kidney SOTRs had received a higher number of immunosuppressants, including antiproliferative agents as well as a combination of ISTs, than liver SOTRs, which may partly explain the higher T-cell induction observed among liver SOTRs. This trend aligned with previous findings from the P304 study in which vaccination elicited more robust humoral responses in liver SOTRs than kidney SOTRs (29). CD4^+^ Th-2 profile in both SOTR and immunocompetent populations was lower in magnitude than Th-1 CD4^+^ response. The stimulation of a Th-1 response in SOTRs following mRNA-1273 vaccination is essential because Th-1 responses are integral for defense against intracellular pathogens and viruses (41). CD8^+^ T-cell responses were lower in magnitude than Th-1 CD4^+^ responses among SOTRs and immunocompetent participants in this study, with this pattern similar to that observed in healthy adults who had received mRNA-1273 and demonstrated robust Th-1-biased CD4^+^ responses with a lower CD8^+^ response (26, 34). Interestingly, mRNA-1273 induced CD8^+^ T-cell responses among kidney SOTRs post dose 3, which was further increased following an additional dose. This result, together with an increase in Th-1 CD4^+^ response in kidney SOTRs, highlights the potential benefit of an additional dose for protection against severe COVID-19 disease in this population.

The ability of polyfunctional T cells to produce multiple pro-inflammatory cytokines (e.g., IFN-γ, IL-2, and TNF-α) in response to activation is predictive of protective immunity (42) and consequently may be used as a measure of activated T-cell responses. Interestingly, polyfunctional responses revealed that both SOTR and immunocompetent participants exhibited Th-1 cytokine signatures with up to five cytokines reported for both groups, with these signatures maintained between primary series vaccination and the additional dose and increased after the additional dose. These findings are of particular interest given that ISTs used in SOTRs are designed to inhibit T-cell immunity.

To our knowledge, there are limited data on cell-mediated immune responses elicited by additional COVID-19 vaccine doses among SOTRs, and this study is among the first clinical trials to evaluate 100-μg mRNA-1273 as an additional dose after the three-dose primary series in SOTR population. Taken together, the results of our study demonstrate the potential benefits of an additional dose of mRNA-1273 to enhance protection in the SOTR population through the induction of CD4^+^ T-cell responses against COVID-19. These findings highlight the importance of the recommendation for additional COVID-19 vaccine doses in this vulnerable population, especially among individuals on complex immunosuppressant regimens (including kidney SOTRs), which is associated with low humoral response or non-response to mRNA vaccination (29, 43–46).

The analysis was limited, as it was an exploratory analysis of a small subset of participants. The net state of immunosuppression for each SOTR, which is an indication of immune status, was not assessed in this study. Additionally, the type of immunosuppressant and the time after transplantation can impact cell-mediated immune responses in SOTRs; analyses of these factors were thus not conducted for this subset of participants. Future studies that build upon this work may benefit from T-cell response data based on the type of immunosuppressant and the time after transplantation. Furthermore, given the urgency of enhancing protection against the Omicron variant, the additional dose was evaluated at a higher 100-μg dose level, as this was the authorized dose at the time of the study, as opposed to the 50-μg dose of the previously authorized original mRNA-1273 vaccine and currently authorized variant-containing vaccine. Assessment of cellular immune responses following an additional 50-μg dose may provide additional data.

In conclusion, a three-dose primary series and an additional dose of 100-μg mRNA-1273 induced SARS-CoV-2-specific CD4^+^ cell-mediated immune responses in the SOTR population. The results from this analysis, together with previous findings from this study, contribute to the currently limited knowledge of humoral and cell-mediated immune responses in SOTRs and demonstrate the importance of additional COVID-19 vaccine doses in this population, which may inform public health and vaccination strategies to aid in reducing the risk of severe COVID-19 disease and mortality for SOTRs.

## Author’s note

Data from this work were presented in a poster presentation format at ECCMID in April 2024.

## Data Availability

Access to patient-level data presented in this article and supporting clinical documents with external researchers who provide methodologically sound scientific proposals will be available upon reasonable request for products or indications that have been approved by regulators in the relevant markets and will be subject to review within 24 months after study completion. Such requests can be made to Moderna Inc., 325 Binney Street, Cambridge, MA 02142, USA; data_sharing@modernatx.com. A materials transfer and/or data access agreement with the sponsor will be required for accessing shared data. All other relevant data are presented in the paper. The protocol is available online at ClinicalTrials.gov: NCT04860297.
