# Supplementary material for: mRNA-1273 COVID-19 vaccine induces CD4+ T-cell responses among solid organ transplant recipients
Source: Front Immunol. 2025 Apr 3;16:1505871. doi: 10.3389/fimmu.2025.1505871 (PMC12004975; doi:10.3389/fimmu.2025.1505871)
Supplement: Supplementary file 1 [file DataSheet1.docx]

***Supplementary Material***

# Supplementary Tables

## Supplementary Table 1. Staining reagents included in the 27-color intracellular cytokine staining flow cytometry panel.

| **Purpose** | **Reagent** | **Clone** | **Manufacturer** | **Catalog number** |
| --- | --- | --- | --- | --- |
| Viability | Viability | NA | Invitrogen | 65-0863 |
| Monocytes | CD14 BUV661 | MΘP9 | BD Biosciences | 741684 |
| B cells | CD19 BUV563 | SJ25C1 | BD Biosciences | 612916 |
| NK cells (FcγR) | CD16 BV570 | 3G8 | Biolegend | 302036 |
| NK cells | CD56 BV750 | 5.1H11 | Biolegend | 362556 |
| T cells | CD3 APC-Fire750 | UCHT1 | Biolegend | 300470 |
|  | CD4 BV480 | SK3 | BD Biosciences | 566104 |
|  | CD8 BUV805 | SK1 | BD Biosciences | 612889 |
| Memory T cells | CCR7 BV605 | G043H7 | Biolegend | 353224 |
|  | CD45RA BUV496 | HI100 | BD Biosciences | 750258 |
| Tregs | CD25 BV650 | M-A251 | BD Biosciences | 563719 |
|  | FOXP3 PE-Cy5.5 | PCH101 | Invitrogen | 35-4776-42 |
| FcγR | CD32 PE-Dazzle594 | FUN-2 | Biolegend | 303218 |
|  | CD64 BV711 | 10.1 | Biolegend | 305042 |
| Th subsets | CXCR3 PE-Cy5 | 1C6/CXCR3 | BD Biosciences | 551128 |
|  | CCR6 BV786 | 11A9 | BD Biosciences | 563704 |
| Th-2 (surface) | CRTh2 PE | BM16 | Biolegend | 350106 |
| Th-1 and CD8 function | IFN-γ V450 | B27 | BD Biosciences | 560371 |
|  | IL-2 APC | MQ1-17H12 | Biolegend | 500310 |
|  | TNF BUV395 | MAb11 | BD Biosciences | 563996 |
| Th-17 | IL-17a PE-Cy7 | BL168 | Biolegend | 512315 |
| Th-2 | IL-4 BB700 | MP4-25D2 | BD Biosciences | Custom |
|  | IL-5 BB630^a^ | TRFK5 | BD Biosciences | Custom |
|  | IL-13 BB630^a^ | JES10-5A2 | BD Biosciences | Custom |
| CD4 function | CD154 BUV737 | TRAP1 | BD Biosciences | 748983 |
| Cytotoxicity | Granzyme B Ax700 | GB11 | BD Biosciences | 560213 |
|  | Perforin FITC | B-D48 | Biolegend | 353310 |
| Activation | Ki67 BB660 | B56 | BD Biosciences | Custom |

APC, antigen presenting cells; CRTh2, chemoattractant receptor homologous molecule expressed on Th-2 cells; FITC, fluorescein isothiocyanate; FcγR, crystallizable fragment with Fcγ receptors; IFN, interferon; IL, interleukin; NA, not applicable; NK, natural killer; PE, phycoerythrin; Th-1, T-helper type 1; Th-17, T-helper type 17; Th-2, T-helper type 2; TNF, tumour necrosis factor.

^a^ IL-5 and IL-13 are detected in the same channel.

## Supplementary Table 2. Response rates for SARS-CoV-2 spike-specific CD4^+^ T cells expressing Th-1 (IFN-γ and/or IL-2), or Th-2 cytokines, or CD8^+^ T cells expressing IFN-γ and/or IL-2 among SARS-CoV-2–seronegative participants who received up to 4 doses (100 µg) of mRNA-1273 in the P304 trial

|  | **Healthy adult** | **SOTR** | |
| --- | --- | --- | --- |
|  |  | **Liver** | **Kidney** |
| **Pre-dose** |  |  |  |
| CD4^+^ IFN-γ and/or IL-2 | 0/12=0% | 0/12=0% | 0/6=0% |
| CD8^+^ IFN-γ and/or IL-2 | 0/12=0% | 0/12=0% | 0/6=0% |
| CD4^+^ Th-2^a^ | 0/12=0% | 0/12=0% | 0/6=0% |
| **Post dose 2** |  |  |  |
| CD4^+^ IFN-γ and/or IL-2 | 11/11=100% | 7/11=64% | 2/5=40% |
| CD8^+^ IFN-γ and/or IL-2 | 4/11=36% | 1/11=9% | 0/5=0% |
| CD4^+^ Th-2^a^ | 1/11=9% | 0/11=0% | 0/5=0% |
| **Post dose 3** |  |  |  |
| CD4^+^ IFN-γ and/or IL-2 | NA | 7/10=70% | 8/15=53% |
| CD8^+^ IFN-γ and/or IL-2 | NA | 2/10=20% | 1/14=7% |
| CD4^+^ Th-2^a^ | NA | 1/10=10% | 0/15=0% |
| **Post booster** |  |  |  |
| CD4^+^ IFN-γ and/or IL-2 | 4/4=100% | 21/29=72% | 34/52=65% |
| CD8^+^ IFN-γ and/or IL-2 | 1/4=25% | 2/29=7% | 9/52=17% |
| CD4^+^ Th-2^a^ | 1/4=25% | 4/29=14% | 5/52=10% |

IFN, interferon; IL, interleukin; NA, not applicable; SOTR, solid organ transplant recipient; Th-1, T-helper type 1; Th-2, T-helper type 2.

^a^Th-2 refers to IL-4, IL-5, and/or IL-13 with CD154 co-expression.
Positive responses were determined using the Fisher’s exact test comparing responses in the peptide pool-stimulation versus the negative control (dimethyl sulfoxide, the peptide diluent).

## Supplementary Figures

## Supplementary Figure 1. Example of the gating strategy of the 27-color intracellular cytokine staining assay.

**
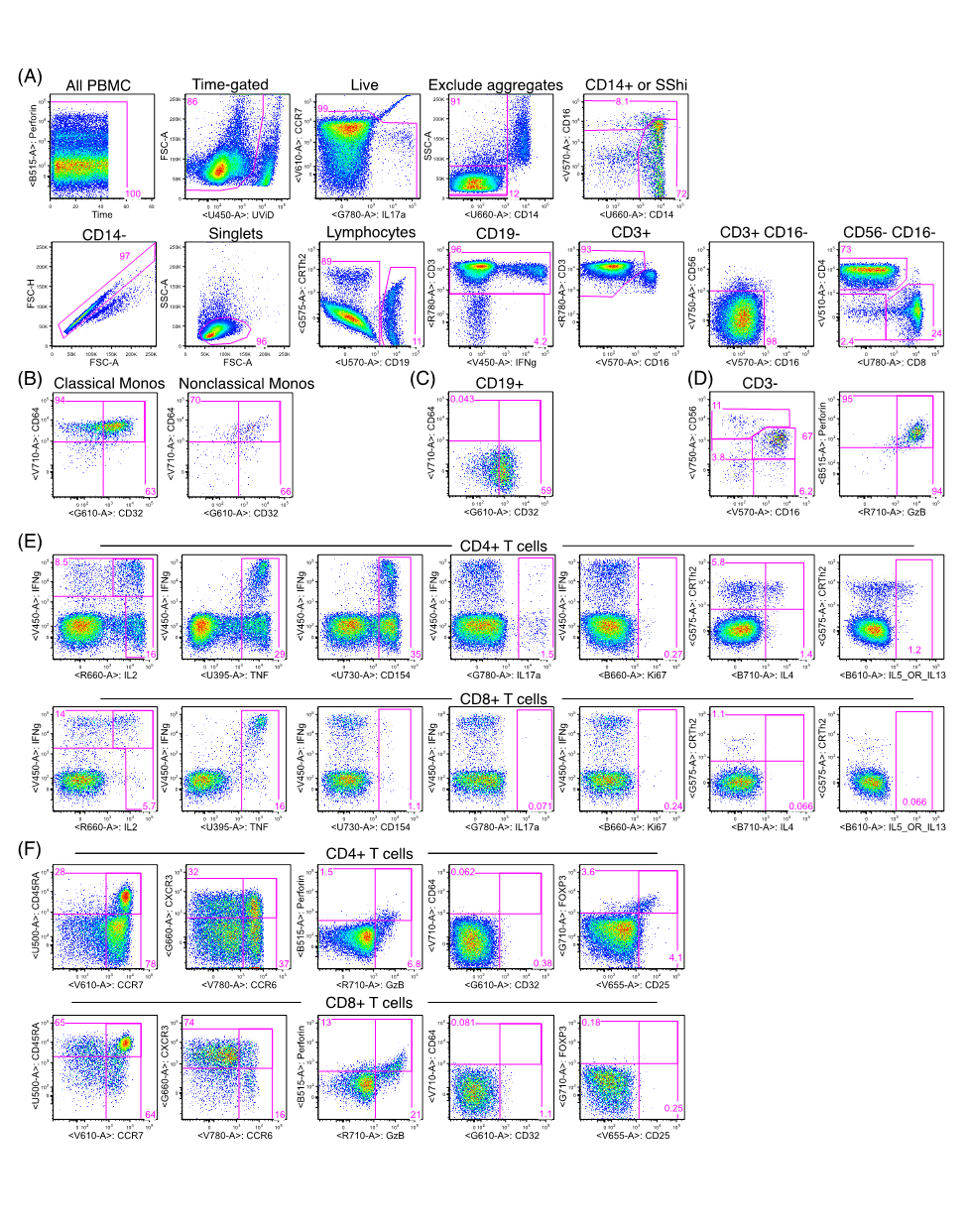
**

Previously cryopreserved PBMC from a healthy adult donor were stimulated with staphylococcal enterotoxin B for 6 hours, stained, and the data were subsequently acquired on a BD FACSymphony instrument. (A) Gating hierarchy to identify lineages. Initial gating on time (seconds) to exclude any events early in collection due to pressure fluctuations, live cell gating, exclusion of aggregates (only 1 plot shown). Monocytes are gated as the inverse of the CD14-SS^lo^ gate, and the upper-right graph shows 2 monocyte subsets based on CD14 versus CD16. Non-monocytes are gated as CD14^-^, followed by singlets, and finally scatter gated on lymphocytes. CD19^+^ cells are gated against CRTh-2 due to the extreme spread of the BUV563 reagent into the G575 detector. CD3^+^/CD3^-^ cells are gated against IFN-γ to ensure that any CD3^+^ cells that have downregulated expression during the stimulation are captured. T cells are further defined as CD3^+^ CD16^-^ on a CD3 versus CD16 plot, followed by gating out CD56^+^ NK T cells on a CD56 versus CD16 plot. Finally, the T cells are further defined by CD4 or CD8 expression on a CD4 versus CD8 plot. (B) CD32 versus CD64 expression of monocyte subsets. (C) CD32 versus CD64 expression on CD19^+^ B cells. (D) NK cell subsets defined by CD16 versus CD56 or perforin versus granzyme B on CD3^-^ lymphocytes. (E) Functional markers for CD4^+^ and CD8^+^ T-cells. A gate is applied for each cytokine, and Boolean gates are created to identify cells expressing different combinations of markers. Most gates are copied, applied to all lineages, and cloned so that any changes to the gate on 1 lineage changes the gate on all lineages. (F) Additional functional and non-functional markers for CD4 and CD8 T cells. As above, these gates are copied and applied to all lineages and clones, apart from CD45RA, due to the difference in expression intensity of this marker on CD8 versus CD4. For some populations, FMO controls were used to set the lower limits of the gates. Some gates are placed higher to improve the specificity, for example, for the functional markers based on the background as observed in the unstimulated controls.

FMO, fluorescence minus one; GzB, granzyme B; IFN, interferon; IL, interleukin; PBMC, peripheral blood mononuclear cells; NK, natural killer; Th-2, T- helper type 2; TNF, tumour necrosis factor.

## **Supplementary Figure 2**. CD8^+^ T-cell polyfunctionality among SARS-CoV-2–seronegative participants using S1+S2 peptide pool in SOTR after 2 and 3 doses of mRNA-1273 in the P304 trial


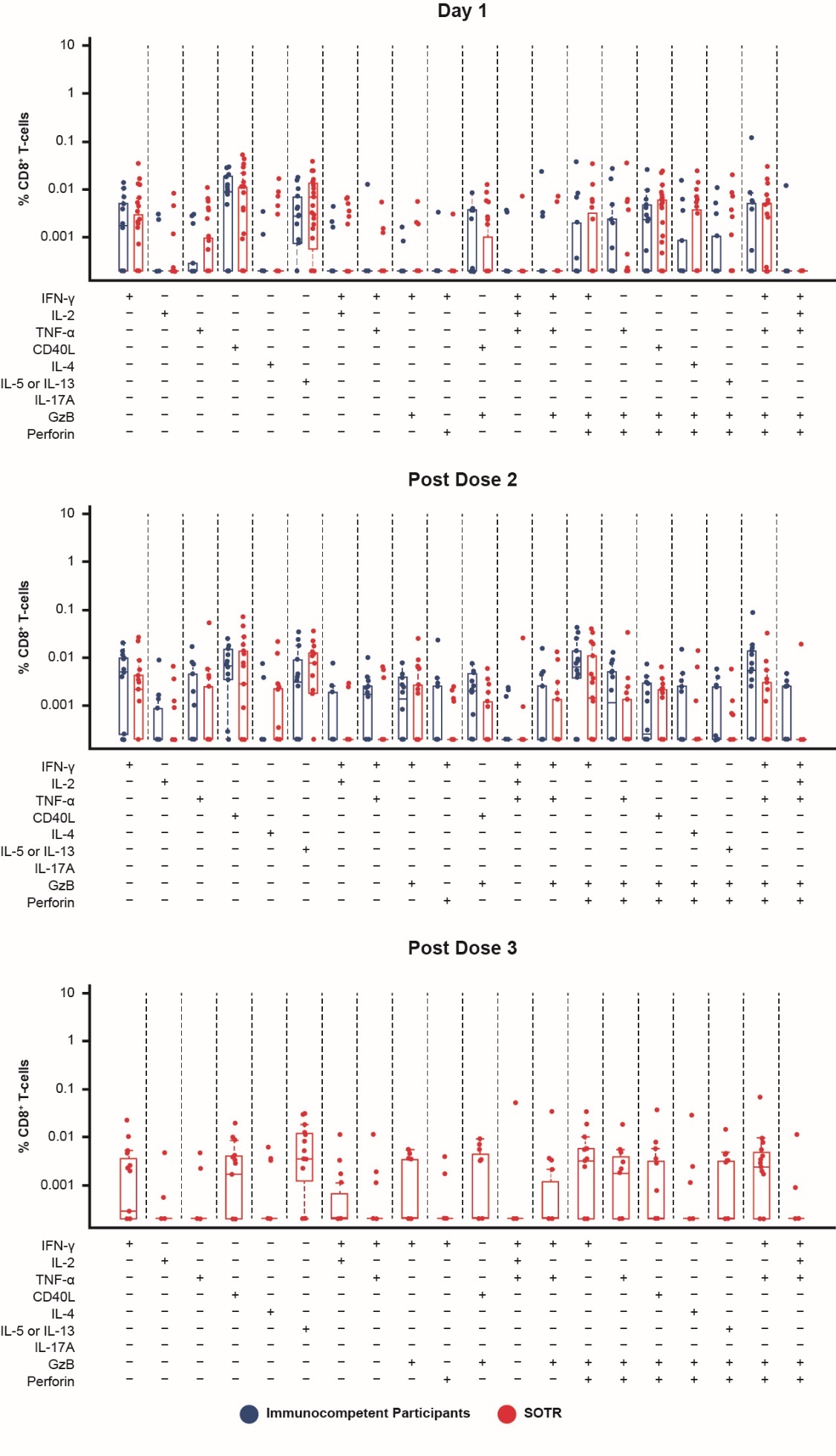


Analysis of polyfunctional CD8^+^ T-cell responses to S1 and S2 peptide pools was conducted using COMPASS (32). Bar graphs represent different cell subsets expressing 1 or more cytokine(s)/functional marker(s). SOTRs (n = 34, red dots) received a 3-dose primary series and additional dose of mRNA-1273 (100-ug); immunocompetent participants (n = 12; blue dots) received a 2-dose primary series and an additional dose of mRNA-1273 (100-µg).

COMPASS, Combinatorial Polyfunctionality Analysis of Antigen-Specific T-cell Subsets; GzB, Granzyme B; IFN-γ, interferon gamma; IL, interleukin; SOTR, solid-organ transplant recipient; TNF-α, tumor necrosis factor alpha.
